# Exact quasi-relativistic wavefunctions of Hydrogen-like atoms

**DOI:** 10.1038/s41598-020-71505-w

**Published:** 2020-09-10

**Authors:** Luis Grave de Peralta

**Affiliations:** 1 Department of Physics and Astronomy, Texas Tech University, Lubbock, TX 79409 USA; 2 Nano Tech Center, Texas Tech University, Lubbock, TX 79409 USA

**Keywords:** Chemical physics, Quantum mechanics

## Abstract

Exact solutions of a novel quasi-relativistic quantum mechanical wave equation are found for Hydrogen-like atoms. This includes both, an exact analytical expression for the energies of the bound states, and exact analytical expressions for the wavefunctions, which successfully describe quantum particles with mass and spin-0 up to energies comparable to the energy associated to the mass of the particle. These quasi-relativistic atomic orbitals may be used for improving ab-initio software packages dedicated to numerical simulations in physical-chemistry and atomic and solid-state physics.

## Introduction

Wavefunctions of Hydrogen-like atoms, which are obtained by solving the Schrödinger equation^1–5^, are often used in ab-initio quantum mechanics simulations^6–8^.

**Figure 1:**
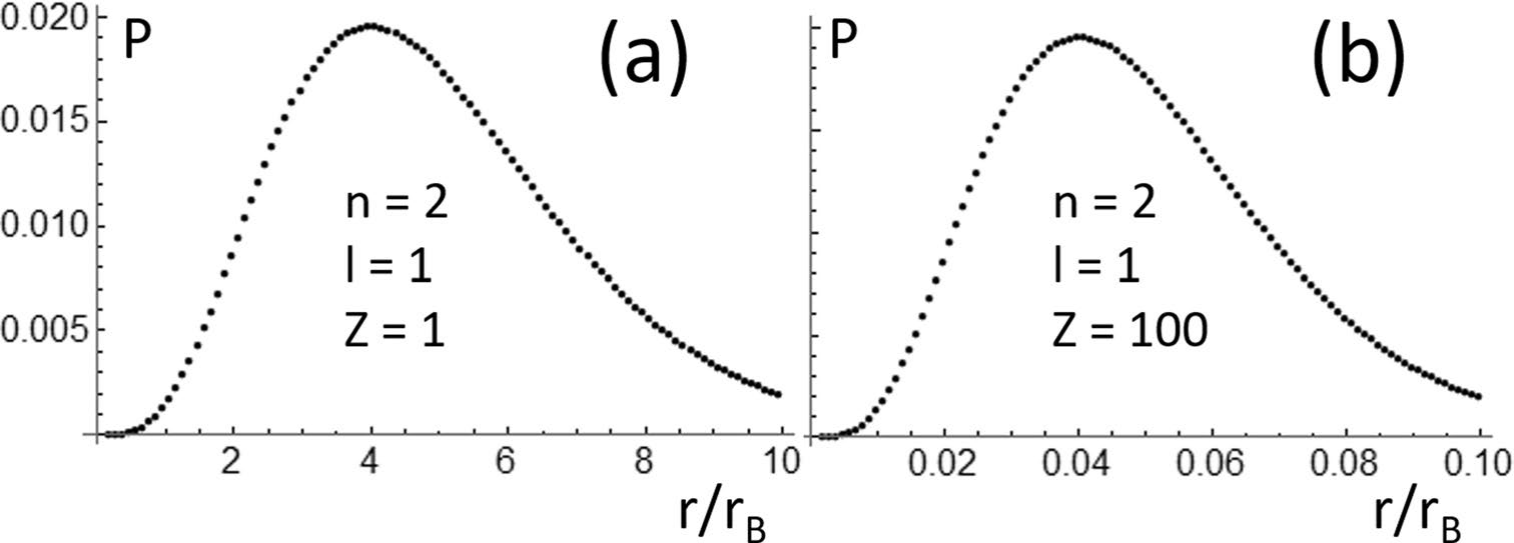
Electron probability functions (*P*(*r*/*r*_*B*_), *r*_*B*_ is the Bohr radius), which were calculated using the solutions of the Schrödinger equation corresponding to *n* = 2 and *l* = 1 for Hydrogen-like atoms with (**a**) *Z* = 1 and (**b**) *Z* = 100.

For instance, there are plotted two probability functions (*P*(*r*)) in Fig. 1. *P*(*r*) were calculated using the following expression:

1 $$ P\left( r \right) = \mathop \int \limits_{r - \Delta r/2}^{r + \Delta r/2} R_{n,l}^{2} \left( \varepsilon \right){\varepsilon^{2}} d\varepsilon . $$

In Fig. 1, *R*_*n,l*_(*r*) = *R*_*2,1,Sch*_(*r*) is the radial part of the solution of the Schrödinger equation for Hydrogen-like atoms^1–5^:

2 $$ i\hbar \frac{\partial }{\partial t}\psi \left( {\overset{\lower0.5em\hbox{$\smash{\scriptscriptstyle\rightharpoonup}$}} {r} ,t} \right) = - \frac{{\hbar^{2} }}{2m}\nabla^{2} \psi \left( {\overset{\lower0.5em\hbox{$\smash{\scriptscriptstyle\rightharpoonup}$}} {r} ,t} \right) + U\left( r \right)\psi \left( {\overset{\lower0.5em\hbox{$\smash{\scriptscriptstyle\rightharpoonup}$}} {r} ,t} \right). $$

In Eq. (2), ℏ is the Plank constant (*h*) divided by 2π, *m* is the mass of the quantum particle, and *U*(*r*) is the Coulomb potential^1–5^:

3 $$ U\left( r \right) = U_{C} \left( r \right) = - \frac{{e^{2} }}{{4\pi \varepsilon_{o} }}\frac{Z}{r} . $$

In Eq. (3)**,** *e* is the electron charge, *Z* is the atomic number, and ε_*o*_ is the electric permittivity of vacuum. *P*(*r*) gives the probability to find the electron inside of a hollow spherical shell of radius *r* and thickness Δ*r*. In both cases (*Z* = 1 and 100), it was assumed that the electron is in a quantum state with principal quantum numbers *n* = 2 and orbital quantum number *l* = 1^1–4^. As seen in Fig. 1, the electron is more closely confined around the nucleus in the Hydrogen-like Fermium atom (*Z* = 100) than in the Hydrogen atom (*Z* = 1). However, when using for simulations wavefunctions obtained by solving the Schrödinger equation, one should be aware of the limitations of this description. The Schrödinger equation is not Lorentz invariant^9^; therefore, it should only be used for atomic simulations when the electron has energies much smaller that the energy associated to his mass^10–13^. The energies of the electron in Hydrogen-like atoms, calculated using the Schrödinger equation, are given by the following expression^1–5^:

4 $$ \k{E}_{n, Sch} = - \left[ {\frac{\mu }{{2\hbar^{2} }}\left( {\frac{{e^{2} }}{{4\pi \varepsilon_{o} }}} \right)^{2} } \right]\frac{{Z^{2} }}{{n^{2} }} . $$

**Figure 2:**
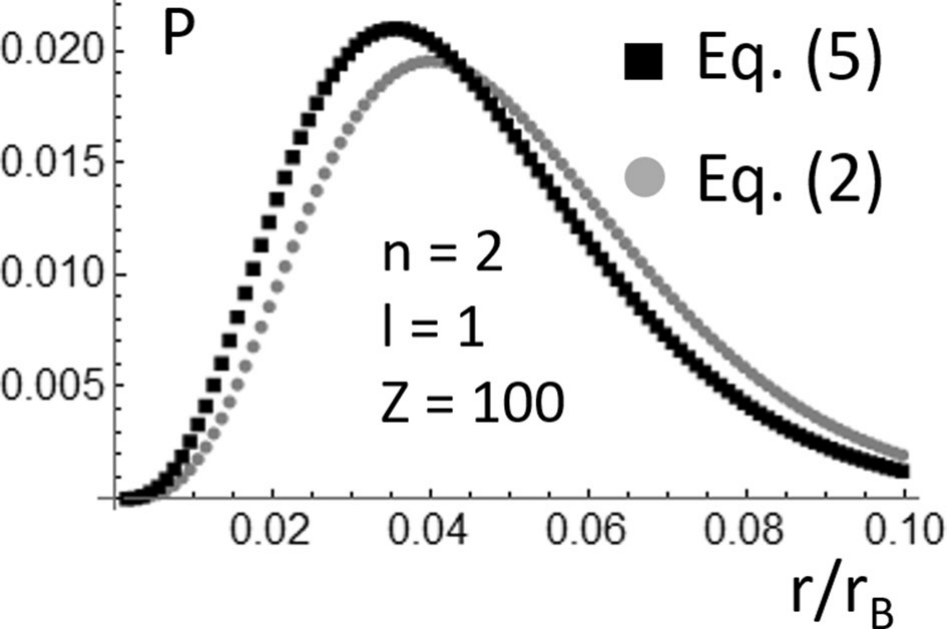
Comparison of the electron probability functions (*P*(*r*/*r*_*B*_), *r*_*B*_ is the Bohr radius) for *n* = 2 and *l* = 1, which were calculated for the Hydrogen-like Fermium atom using the solutions of the Schrödinger equation and the quasi-relativistic wave equation.

In Eq. (4), μ = (*m*_*e*_*m*_*n*_)/(*m*_*e*_ + *m*_*n*_) is the electron’s reduced mass, and *m*_*e*_ and *m*_*n*_ are the electron and nucleus masses, respectively. Using Eq. (4) and denoting with the symbol (*c*) the speed of the light in vacuum, one can then find that |*Ę*_*2,Sch*_|/μc^2^ ~ 10^–5^ and ~ 0.0666 for *Z* = 1 and 100, respectively. Consequently, one should expect that the probability function for *Z* = 1 plotted in Fig. 1 is a better approximation to reality than *P*(*r*) for *Z* = 100. This expectation is confirmed by the probability functions plotted in Fig. 2, where the probability function shown in Fig. 1b is superposed to the corresponding probability function calculated using the solutions of the following recently reported quasi-relativistic wave equation^10–13^:

5 $$ i\hbar \frac{\partial }{\partial t}\psi \left( {\overset{\lower0.5em\hbox{$\smash{\scriptscriptstyle\rightharpoonup}$}} {r} ,t} \right) = - \frac{{\hbar^{2} }}{{\left[ {\gamma_{V} \left( r \right) + 1} \right]m}}\nabla^{2} \psi \left( {\overset{\lower0.5em\hbox{$\smash{\scriptscriptstyle\rightharpoonup}$}} {r} ,t} \right) + U\left( r \right)\psi \left( {\overset{\lower0.5em\hbox{$\smash{\scriptscriptstyle\rightharpoonup}$}} {r} ,t} \right). $$

Formally, Eq. (5) can be obtained by substituting 2* m* in Eq. (2) by (*γ*_*V*_ + 1)*m*. The factor *γ*_*V*_ is found in many equations of the Einstein’s special theory of relativity, and depends on the ratio between the square of the particle’s speed (*V*^2^) and *c*^2^^14–16^:

6 $$ \gamma_{V} = \frac{1}{{\sqrt {1 - \frac{{V^{2} }}{{c^{2} }}} }} . $$

The quasi-relativistic wave equation (Eq. 5) successfully describes a particle of mass *m* moving at quasi-relativistic energies (*Ę* = *K* + *U* ~ *mc*^2^)^10–13^. Equation (5) implies that the relation between the particle’s kinetic energy (*K*) and its linear momentum (*p*) is the one required by the special theory of relativity^10–16^

7 $$ K = \frac{{p^{2} }}{{\left( {\gamma_{V} + 1} \right)m }} . $$

This contrasts with the non-relativistic relation, *K* = *p*^2^/2* m*, for a particle described by the Schrödinger equation^1–5,10–16^. It should be noted that everywhere in this work, *Ę* = *K* + *U* is called the energy or the quasi-relativistic energy of the particle. *Ę* is not the total relativistic energy of the particle (*E*), which is given by the following expression: *E* = *Ę* + *mc*^*2*^^10–16^*.* Also, this work focuses on Hydrogen-like atoms; therefore, *m* = μ in Eqs. (2), (5), and (7). The electron energy when the Hydrogen’s electron is in the quantum state *n* = 2, *l* = 1 is much smaller than μc^2^. Consequently, the probability functions calculated using Eqs. (2) and (5) superpose to each other almost perfectly (not shown). However, as shown in Fig. 2, this does not happen for Z = 100. *Ę*_*2,Sch*_ ~ − 0.0666 μc^2^ in the Hydrogen-like Fermium atom; at these energies, as shown in Fig. 2, the Schrödinger equation underestimate the confinement of the electron around the Fermium nucleus. The wavefunctions found solving Eq. (5) then allows for improving the calculation of *P*(*r*) at quasi-relativistic electron energies.

Previously, the quasi-relativistic wave equation has been solved, following the same mathematical steps required for solving the same problems using the Schrödinger equation, for a free particle^10^, confinement of a quantum particle in box^10,12,13^, reflection by a sharp quantum potential^12^, tunnel effect^12^, and the Hydrogen atom^11,13^. In this work, it is discussed how to find the quasi-relativistic wavefunctions which are solution of Eq. (5). We compare the quasi-relativistic wavefunctions found with the corresponding ones for the Schrödinger equation. It is shown that, due to the high similitude between Eqs. (2) and (5), exact analytical solutions of Eq. (5) can be found. Moreover, this can be done using the same mathematical techniques used for solving the Schrödinger equation and with no more difficulty. In atoms and molecules, the number of particles is constant. This is because the energies of the electrons in atoms and molecules are smaller than the energy associated to the electron’s mass. The energies of the external electrons in atoms and molecules are non-relativistic; therefore, the wavefunctions calculated solving the Schrödinger equation are adequate for conducting simulations involving these electrons. However, the internal electrons in heavy atoms have quasi-relativistic energies; therefore, the quasi-relativistic wavefunctions, which are discussed for the first time in this work, can be used for improving ab-initio quantum mechanics simulations involving the inner electrons of heavy atoms. This also can be done using the exact relativistic wavefunctions obtained solving the Dirac equation^2,15,16^. However, the Dirac equation and the Dirac’s (bispinor) wavefunctions are much more complex than Eq. (5) and its (scalar) wavefunctions^2,15–17^. Both, Eq. (5) and the Schrödinger equation, allow building a relatively simple and intuitive quantum theory of atoms and molecules, where the number of electrons is constant, and no positrons are involved. However, Eq. (5) provides the advantage of including the correct relation between *K* and *p*, without paying a heavy price in mathematical and theoretical complexity. In addition, the wavefunctions of Eq. (5) may be smoothly introduced in general courses of Quantum Mechanics for illustrating the consequences, for the quantum theory, resulting from the introduction on it of the basic ideas of the special theory of relativity. It should be remarked that the wavefunction in Eq. (5) is a scalar. This is because Eq. (5) does include the correct relativistic relation between *K* and *p*^11,13^, but does not include the electron spin. The Dirac equation includes exactly both the electron spin and the relativistic effects. This requires a bispinor wavefunction with 4 components^2,15–18^. However, there are approximated theories only requiring, for the description of the spin effects, spinor two-component wavefunctions^2,15–23^. In this work, the attention is focused on the consequences resulting, from including the correct relativistic relation between *K* and *p*, for the quantum theory of Hydrogen-like atoms. The rest of this paper is organized in the following way. In the next section, for self-reliance purposes, a summary about solving Eq. (5) is presented. In addition, and for the first time, an equation given the exact analytical expression of the energy of the bounded states in Hydrogen-like atoms is presented. Then, in the following section, the analytical expressions of several quasi-relativistic wavefunctions are presented and compared with the corresponding wavefunctions for Eq. (2). Finally, the conclusions of this work are given in the “Conclusions”.

## Solving the quasi-relativistic wave equation

Due to the high similitude between Eqs. (2) and (5), exact analytical solutions of Eq. (5), with *U*(*r*) given by Eq. (3), can be found following the same procedures needed for solving the Schrödinger equation for Hydrogen-like atoms^1,4,11^. Expressing in spherical coordinates the Laplace operator in Eq. (5) and looking for separated variables solutions of Eq. (5)^1,4,11^:

8 $$ \psi \left( {r,\theta ,\varphi ,t} \right) = \frac{\chi \left( r \right)}{r}\Omega \left( {\theta ,\varphi } \right)e^{{\frac{i}{\hbar}\k{E}t}} . $$

Results^1,4,11^

$$ \Omega_{l,m} \left( {\theta ,\varphi } \right) = Y_{l}^{\left( m \right)} \left( {\theta ,\varphi } \right) ; $$

9 $$ \eta = l\left( {l + 1} \right); \quad l = 0, 1, 2 \ldots ;\quad m = - l, - l + 1, \ldots 0, 1, \ldots ,l. $$

where *Y*_*l*_^*(m)*^ are the spherical harmonic functions^1–5^. And:

10 $$ \frac{{d^{2} }}{{dr^{2} }}\chi \left( r \right) + \frac{{\left[ {\gamma_{V} \left( r \right) + 1} \right]\mu }}{{\hbar^{2} }}\left[ { \k{E}- W_{C} \left( r \right)} \right]\chi \left( r \right) = 0 . $$

In Eq. (10):

11 $$ W_{C} \left( r \right) = \left[ {U_{C} \left( r \right) + \frac{{\hbar^{2} }}{{\left[ {\gamma_{V} \left( r \right) + 1} \right]\mu }}\frac{{l\left( {l + 1} \right)}}{{r^{2} }}} \right]. $$

When the electron moves slowly (*V*^2^ <  < *c*^2^) then γ_*V*_ ~ 1; therefore, Eq. (10) reduces to the radial equation for hydrogen-like atoms obtained using the Schrödinger equation^4^. If one wants to be able to solve Eq. (10), using the same techniques that are used for solving the Schrödinger’s radial equation for a hydrogen-like atoms, it is necessary to eliminate γ_*V*_ from Eq. (11) This can be done using the relativistic equation^11,14^:

12 $$ K = \left( {\gamma_{V} - 1} \right)\mu c^{2} . $$

Therefore:

13 $$ \frac{{\left[ {\gamma_{V} \left( r \right) + 1} \right]\mu }}{{\hbar^{2} }} = \frac{{K + 2\mu c^{2} }}{{c^{2} \hbar^{2} }} = \frac{{\left[ {\k{E} - U_{C} \left( r \right)} \right] + 2\mu c^{2} }}{{c^{2} \hbar^{2} }} . $$

One can then use Eq. (13) and introduce the following variables^11^:

14 $$ \rho \equiv \zeta r, \quad \rho_{o} \equiv \left( {\frac{{\mu e^{2} }}{{2\pi \varepsilon_{o} \hbar^{2} \zeta }} - \alpha \frac{\hbar \zeta }{{\mu c}}} \right)Z , \quad\rho_{1} \equiv \left[ {1 - \left( {\frac{\hbar \zeta }{{2\mu c}}} \right)^{2} } \right]. $$

In Eq. (14), α is the fine-structure constant^15,16^:

15 $$ \alpha = \frac{1}{{4\pi \varepsilon_{o} }} \frac{{e^{2} }}{{\hbar {\varvec{c}}}} \sim 1/137 . $$

And^4,11^:

16 $$  \zeta = \frac{1}{\hbar }\sqrt {- 2\mu \k{E} } . $$

This allows for rewriting Eq. (10) in the following way^11^:

17 $$ \frac{{d^{2} }}{{d\rho^{2} }}\chi \left( \rho \right) = \left[ {\rho_{1} - \frac{{\rho_{o} }}{\rho } + \frac{{l\left( {l + 1} \right) - \alpha^{2} Z^{2} }}{{\rho^{2} }}} \right] \chi \left( \rho \right). $$

When ℏζ <  < μ*c* and α^2^Z^2^ <  < 1, Eq. (17) reduces to the equation that is solved for the Hydrogen atom when using the Schrödinger equation^4^:

18 $$ \frac{{d^{2} }}{{d\rho^{2} }}\chi \left( \rho \right) = \left[ {1 - \frac{{\rho_{o} }}{\rho } + \frac{{l\left( {l + 1} \right)}}{{\rho^{2} }}} \right] \chi \left( \rho \right). $$

From Eq. (18) can be found that^4^:

19 $$ \rho_{o} = 2n,\quad n = 1, 2, 3, \ldots $$

Consequently, when ℏζ <  < μ*c* and α^2^Z^2^ <  < 1, Eq. (4) can be obtained from Eqs. (14), (16), and (19)^4^. However, each of the three terms in the right side of Eq. (17) contains a different quasi-relativistic correction to the radial equation of Hydrogen-like atoms. Fortunately, the quasi-relativistic Eq. (17) can be solved as Eq. (18) was solved^4,11^. One can look for a solution of Eq. (17) of the following form^11^:

20 $$ \chi \left( \rho \right) \equiv \tau \left( \rho \right) \rho^{{\frac{ 1}{2}\left[ {1 + \sqrt {\left( {1 + 2l} \right)^{2} - 4\alpha^{2} Z^{2} } } \right]}} e^{{ - \sqrt {\rho_{1} } \rho }} . $$

This allows expressing τ(ρ) as a finite power series in ρ^4,11^:

21 $$ \tau \left( \rho \right) = \mathop \sum \limits_{j = 0}^{{j_{\max } }} a_{j} \rho^{j} . $$

In Eq. (21), *j*_*max*_ = *n* − (*l* + 1) and^11^:

22 $$ a_{j + 1} = \frac{{\sqrt {\rho_{1} } \left[ {2j + \left( {1 + \sqrt {\left( {1 + 2l} \right)^{2} - 4\alpha^{2} Z^{2} } } \right)} \right] - \rho_{o} }}{{\left( {j + 1} \right)\left[ {j + \left( {1 + \sqrt {\left( {1 + 2l} \right)^{2} - 4\alpha^{2} Z^{2} } } \right)} \right]}}a_{j} . $$

Evaluating Eq. (22) for *j* = *j*_*max*_ and making *a*_*jmax*+1_ = 0, one can obtain^11^:

23 $$ \rho_{o} = \left[ {2n + \Delta \left( {l,Z} \right)} \right]\sqrt {\rho_{1} } . $$

In Eq. (23)^11^:

24 $$ \Delta \left( {l,Z} \right) = \left[ {\left( {1 + \sqrt {\left( {1 + 2l} \right)^{2} - 4\alpha^{2} Z^{2} } } \right) - 2\left( {l + 1} \right)} \right] , \sqrt {\left( {1 + 2l} \right)^{2} - 4\alpha^{2} Z^{2} } \sim \left( {1 + 2l} \right) - \frac{{2\alpha^{2} Z^{2} }}{{\left( {1 + 2l} \right)}} - \frac{{2\alpha^{4} Z^{4} }}{{\left( {1 + 2l} \right)^{3} }}. $$

In some cases, for heavy Hydrogen-like atoms with Z >  > 1, the term inside the square root in Eq. (24) could be negative; in these cases, the approximation to the square root included in Eq. (24) should be used. Substituting ρ_o_ and ρ_1_ given by Eq. (14) in Eq. (23), solving the resulting equation for ζ, and using Eq. (16), produce an exact analytical expression for Ȩ, which now depends not only on the principal quantum number *n*, but also on the orbital quantum number *l* and *Z*^11^. This expression is given here for the first time:

25 $$ \k{E}_{n,l} = - \frac{{\mu c^{2} }}{\Xi }\left[ {\Xi - \left( {2n + \Delta } \right)\sqrt \Xi } \right]. $$

In Eq. (25), Δ = Δ(*l*, *Z*) given by Eq. (24), and Ξ is given by the following expression :

26 $$ \Xi = 4n^{2} + 4\alpha^{2} Z^{2} + 4n\Delta + \Delta^{2} . $$

Expressing Eq. (25) as a series in powers of *α,* and taking the first terms of the series up to *α*^4^, conduct exactly to the following approximated expression of Eq. (25):

27 $$ \k{E}_{n,l} = \k{E}_{n, Sch} \left\{ {1 - \frac{{\alpha^{2} Z^{2} }}{{n^{2} }}\left[ {\frac{3}{4} - \frac{n}{l + 1/2}} \right]} \right\}. $$

In Eq. (27), *Ę*_*n,Sch*_ given by Eq. (4) was rewritten as a function of α in the following way:

28 $$ \k{E}_{n, Sch} = - \frac{{\mu c^{2} \alpha^{2} Z^{2} }}{{2n^{2 } }}. $$

Therefore, as should be expected, when α^2^Z^2^/*n*^2^ <  < 1, Eq. (25) reduces to Eq. (4). Moreover, Eq. (27) is exactly equal to the relativistic correction to the kinetic energy in first-order perturbation theory^4,17^. A comprehensive comparison between the energies calculated using Eq. (2), Eq. (5), and the available experimental data corresponding to the Hydrogen’s spectrum, was recently reported^11,17^. In that work, Eq. (25) was not used but the following approximate equation^11^:

29 $$ \k{E}_{n, l} = - \left[ {\frac{\mu }{{2\hbar^{2} }}\left( {\frac{{e^{2} }}{{2\pi \varepsilon_{o} }}} \right)^{2} } \right]\frac{{Z^{2} }}{{\left[ {2n + \Delta \left( {l,Z} \right)} \right]^{2} }} . $$

Equation (29) was obtained assuming that the quasi-relativistic corrections included in ρ_o_ and ρ_1_ do not need to be accounting for because they are too small; therefore, Eq. (29) only includes the effect of the quasi-relativistic correction included in the centrifugal term in Eq. (17)^11^.

**Figure 3:**
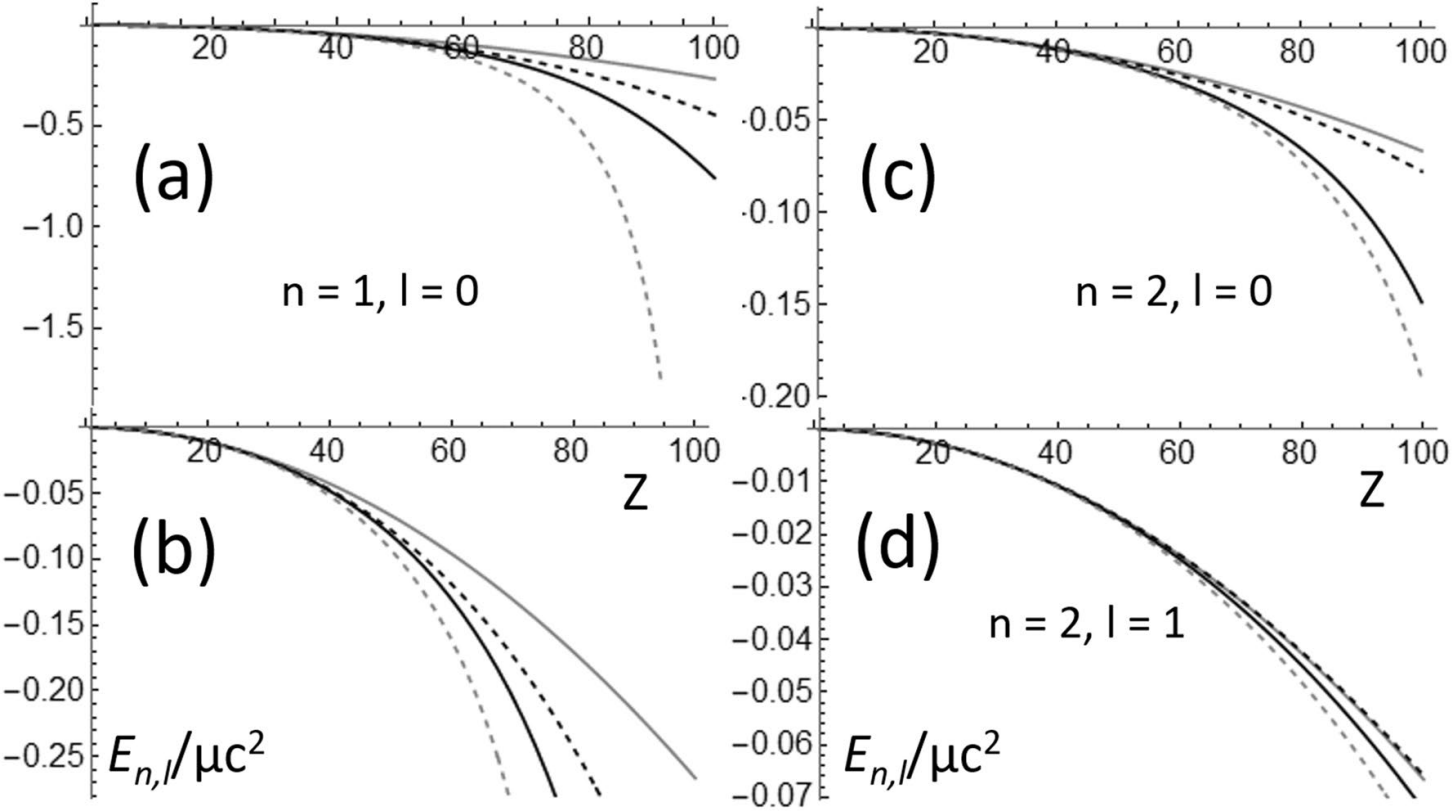
Comparison of the dependence on *Z* of the calculated energies for (**a**,**b**) *n* = 1 and *l* = 0, (**c**) *n* = 2 and *l* = 0, and (**d**) *n* = 2 and *l* = 1. *Ę*_*n,l*_ was evaluated using Eqs. (gray continuous) (28), (black dashed) (27), (black continuous) (25), (gray dashed) (29).

A comparison of *Ę*_*n,l*_ dependence on *Z*, when *Ę*_*n,l*_ is calculated using Eqs. (25), and (27–29), is shown in Fig. 3. In all cases the Schrödinger equation (Eqs. 4 and 28, gray continuous curves in Fig. 3) gives the smaller value of |*Ę*_*n,l*_|, while Eq. (29) gives the largest (gray dashed curves). As expected, all equations give similar values when |*Ę*_*n,l*_|/μc^2^ <  < 1. Interestingly, at quasi-relativistic energies Eq. (27), the well-known equation given the relativistic correction to the kinetic energy in first-order perturbation theory (black dashed curves) underestimate the exact value of |*Ę*_*n,l*_| (Eq. 25), black continuous curves in Fig. 3. It is worth restating Eq. (25) is an exact result presented here for the first time, while Eq. (27) is a well-known approximated result^4,17^. This strongly supports the use of the Grave de Peralta equation (this is how the author proposes Eq. (5) to be called) for describing quantum particles with mass and spin-0 moving at quasi-relativistic energies^10–13^. A comparison between the energies calculated using Eq. (25), and the energy values calculated using the Dirac equation, allows a precise determination of which energy contributions are included in Eq. (25) and which ones are not included on it. The following equation gives the exact energies calculated using the Dirac equation^2^:

30 $$ \k{E}_{n,l,j} = \mu c^{2} \left[ {1 + \left( {\frac{Z\alpha }{{n - \left( {j + \frac{1}{2}} \right) + \sqrt {\left( {j + \frac{1}{2}} \right) - Z^{2} \alpha^{2} } }}} \right)^{2} } \right]^{{ - \frac{1}{2}}} - \mu c^{2} . $$

**Figure 4:**
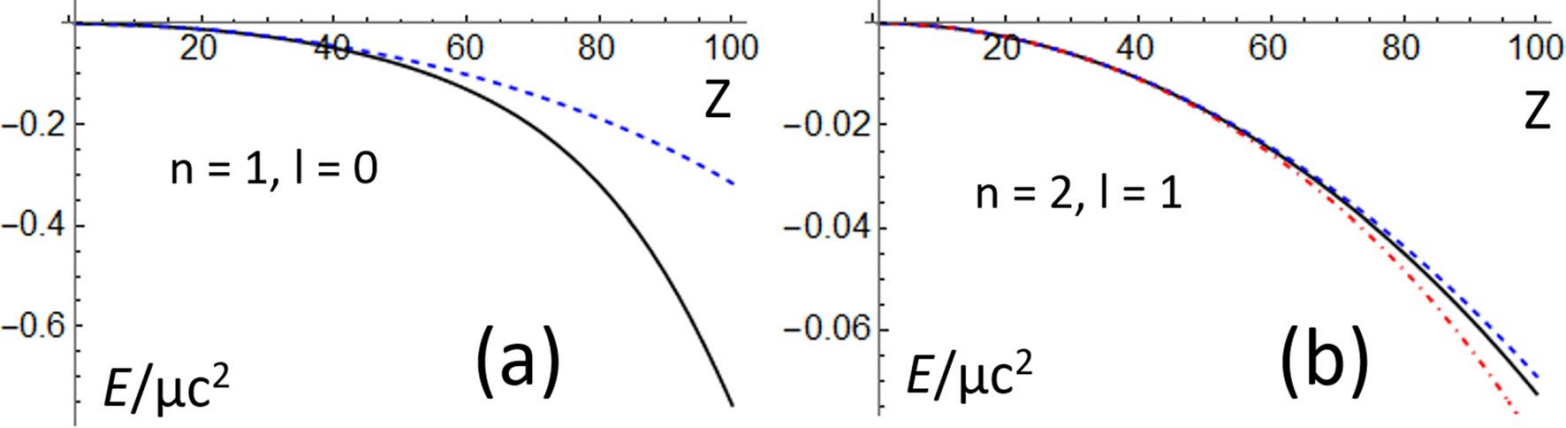
Comparison of the dependence on *Z* of the calculated energies for (**a**) *n* = 1 and *l* = 0, (**b**) *n* = 2 and *l* = 1. *Ę* was evaluated using Eqs. (black continuous) (25), (blue dashed) (30) with *j* = *l* + ½, and (red dot-dashed) (30) with *j* = *l*—½.

In Eq. (30), *j* = *l* ± ½^2^. Figure 4, shows a comparison of the energies calculated using Eqs. (25) and (30).

The Dirac’s energies include three corrections to the energies calculated using the Schrödinger equation^2,17^. The first is the relativistic correction to the kinetic energy. This correction is exactly included in Eq. (25)^11,18^. The second is the so-called Darwin correction, which is only non-zero when *l* = 0^2,11,17,18^. The Darwin correction is related with the non-zero probability for the electron to be found in the nucleus when *l* = 0^2,17^. The third correction is the spin–orbit correction, which is only non-zero when *l* > 0^2,11,17,18^. As shown in Fig. 4a, the Darwin correction destabilizes the electron (*Ę*_*n,l,j*_ > *Ę*_*n,l*_)^17^. This destabilization increases at quasi-relativistic energies (Z >  > 1). As shown in Fig. 4b, the spin–orbit correction splits the quasi-relativistic energy level *Ę*_*n,l*_ in two energy levels corresponding to *j* = *l* ± ½^2,17^. The spin–orbit correction also increases when Z >  > 1. However, the energy difference |*Ę*_*n,l,j*_—*Ę*_*n,l*_| is smaller in Fig. 4b than in Fig. 4a because |*Ę*| is an order of magnitude larger in the ground state with *n* = 1 and *l* = 0 than in the excited state with *n* = 2 and *l* = 1.

## Quasi-relativistic wave functions

The exact analytical wavefunctions of Eq. (5) are given by Eq. (8) with *Ę*_*n,l*_ given by Eq. (25), *Ω*_*l,ml*_ by Eq. (9), and χ(ζ*r*) and τ(ζ*r*) given by Eqs. (20–21) and (16). Therefore, the quasi-relativistic wavefunctions only differ from the wavefunctions of the Schrödinger equation in the values of *Ę* and in the radial part of the wavefunctions. τ(ρ) is given by Eq. (21) with *j*_*max*_ = *n* − (*l* + 1). Before using Eq. (22) for finding the coefficients, *a*_*j*_, ρ_1_ should be determined using Eqs. (14), (16), and (25), then ρo can be obtained using Eqs. (23) and (24). After τ(ρ) is found for given values of *n* and *l*, χ(ρ) can be obtained using Eq. (20). Finally, the complete unnormalized radial wavefunction is *Я*_n,l_ = χ(ζ*r*)/*r*. Therefore, the normalized radial wavefunctions are given by the following equation:

31 $$ {{R}}_{{n,l}} \left( r \right) = \frac{1}{\beta }{{\reflectbox{R}}_{{n,l}}} \left( r \right),\quad\beta ^{2}  = \mathop \int \nolimits_{0}^{{ + \infty }}  {\reflectbox{R}}^{2} \left( \varepsilon  \right)\varepsilon ^{2} d\varepsilon . $$

The above description constitutes a general method for obtaining any radial wavefunctions corresponding to Eq. (5). In what follows, for illustration purposes, the *Я*_*n,l*_(*r*) functions corresponding to the ground state and first excited states of Hydrogen-like atoms will be explicitly discussed. In addition, *P*(*r*) will be calculated using Eq. (1) for doing a meaningful comparison (Fig. 2) between *R*_*n,l*_(*r*) and the corresponding radial wavefunctions of the Schrödinger equation^4^. Following the general method stated above, it was obtained the following expression for the ground state (*n* = 1, *l* = 0) of Hydrogen-like atoms:

32 $$ {{\reflectbox{R}}_{1,0}} \left( r \right) \propto \frac{{\left( {{C_{1,0}} \frac{\mu cr}{\hbar }} \right)^{{B_{1,0} }} }}{r}e^{{ - A_{1,0} \frac{\mu cr}{\hbar }}} . $$

In Eq. (32), *A*_1,0_, *B*_1,0_, and *C*_1,0_ are given by the following expressions:

33 $$ A_{1,0} = \frac{{\sqrt {2 - 2\Delta } }}{2}, \quad B_{1,0} = \frac{1 + \Delta }{2} , \quad C_{1,0} = \sqrt {2 - \sqrt {2 + 2\Delta } } . $$

In Eq. (33), Δ = Δ(*l*, *Z*) given by Eq. (24); therefore, by expressing the parameters *A*_1,0_, *B*_1,0_, and *C*_1,0_ as a series in powers of α each, and approximating them by the first terms of the series, one can obtain the following approximations for this parameters, which are valid when α^2^ *Z*^2^ <  < 1:

34 $$ A_{1,0} \sim \alpha Z, B_{1,0} \sim 1 , C_{1,0} \sim \alpha Z. $$

Consequently, *Я*_1,0_(*r*) ~ *Я*_1,0*,Sch*_(*r*) when α^2^ *Z*^2^ <  < 1^4^:

35 $$ {{\reflectbox{R}}} _{{1,0,Sch}} \left( r \right) \propto \frac{Z}{{r_{B} }}e^{{ - \frac{{Zr}}{{r_{B} }}}}   . $$

In Eq. (35), *r*_*B*_ = ℏ/(*αμc*) is the Bohr radius^1–5^. There are two possible values of *l* in the first excited state (*n* = 2); *l* = 0 and *l* = 1^1–5^. For *l* = 0, it was obtained:

36 $$ {{\reflectbox{R}}}_{{2,0}} \left( r \right) \propto \frac{{\left( {C_{{2,0}} \frac{{\mu cr}}{\hbar }} \right)^{{B_{{2,0}} }} }}{{2B_{{2,0}}r}}\left[ {2B_{{2,0}}  - 2A_{{2,0}} \frac{{\mu cr}}{\hbar }} \right]e^{{ - A_{{2,0}} \frac{{\mu cr}}{\hbar }}} . $$

In Eq. (36):

37 $$ A_{2,0} = \frac{\sqrt 2 Z}{{\sqrt {5 + 3\Delta } }}, \quad B_{2,0} = \frac{1 + \Delta }{2} , \quad C_{2,0} = \sqrt {\frac{{10 + 6\Delta - \sqrt 2 \eta \sqrt {5 + 3\Delta } - 3\sqrt {10 + 6\Delta } }}{5 + 3\Delta }} . $$

Following the same procedure discussed above, it can be shown that *Я*_2,0_(*r*) ~ *Я*_2,0,*Sch*_(*r*)^4^:

38 $$ {{\reflectbox{R}}} _{{2,0,Sch}} \left( r \right) \propto \left( {\frac{Z}{{2r_{B} }} - \frac{{Z^{2} r}}{{4r_{B} ^{2} }}} \right)e^{{ - \frac{{Zr}}{{2r_{B} }}}} . $$

Finally, it was obtained for *n* = 2 and *l* = 1:

39 $$ {{\reflectbox{R}}}_{{2,1}} \left( r \right) \propto \frac{{\left( {C_{{2,1}} \frac{{\mu cr}}{\hbar }} \right)^{{B_{{2,1}} }} }}{{r}}e^{{ - A_{{2,0}} \frac{{\mu cr}}{\hbar }}} . $$

In Eq. (39):

40 $$ A_{2,1} = \frac{\sqrt 2 Z}{{\sqrt {5 + \Delta } }}, B_{2,1} = \frac{1 + \Delta }{2},\,C_{2,1} = \sqrt {\frac{{10 + 2\Delta - \sqrt 2 \Delta \sqrt {5 + \Delta } - \sqrt {10 + 2\Delta } }}{5 + \Delta }} . $$

Here again, *Я*_*2,1*_(*r*) ~ *Я*_*2,1,Sch*_(*r*) when α^2^*Z*^2^ <  < 1^4^:

41 $$ {{\reflectbox{R}}} _{{2,1,Sch}} \left( r \right) \propto \frac{{Z^{2} r}}{{4r_{B} ^{2} }}e^{{ - \frac{{Zr}}{{2r_{B} }}}}.  $$

**Figure 5:**
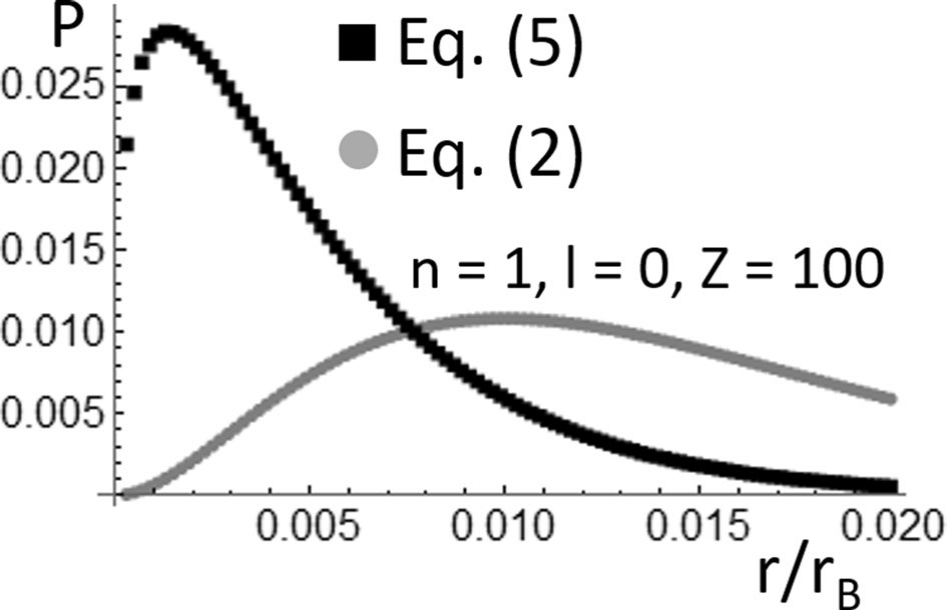
Comparison of the electron probability functions (*P*(*r*/*r*_*B*_), *r*_*B*_ is the Bohr radius) for *n* = 1 and *l* = 0, which were calculated for the Hydrogen-like Fermium atom using the solutions of the Schrödinger equation and the quasi-relativistic wave equation.

As shown in Fig. 3a,b, there is a notable difference between the energy *Ę*_*1,Sch*_ ~—0.2663 μc^2^ corresponding to the ground state of the Hydrogen-like Fermium atom (*Z* = 100), which is calculated using the Schrödinger equation (Eqs. 4 and 28), and the energy *Ę*_*1,0*_ ~—0.7556 μc^2^ which is calculated using Eq. (25). At energies like *Ę*_*1,0*_ ~—0.7556 μc^2^, one should expect a notable difference between *Я*_*1,0*_(*r*) and *Я*_*1,0,Sch*_(*r*). This is confirmed by the probability functions (*P*(*r*/*r*_*B*_) shown in Fig. 5, which were calculated using Eqs. (1) and (31) and the functions *Я*_*1,0*_(*r*) and *Я*_*1,0,Sch*_(*r*) given by the Eqs. (32) and (35), respectively. Clearly, using the Schrödinger equation causes a notable underestimation of the confinement of the electron around the Fermium nucleus. This result was previously mentioned in the preliminary discussion of Fig. 2 made in the Introduction. The probability functions (*P*(*r*/*r*_*B*_) shown in Fig. 2 were calculated using Eqs. (1) and (31) and the functions *Я*_*2,1*_(*r*) and *Я*_*2,1,Sch*_(*r*) given by the Eqs. (39) and (41), respectively. Using Eq. (25) with *n* = 2, *l* = 1, and *Z* = 100, one can find that *Ę*_*2,1*_ ~ − 0.0724 μc^2^; therefore |*Ę*_*1,0*_| is an order of magnitude larger than |*Ę*_*2,1*_|. A comparison of Figs. 2 and 5 reveals that the underestimation of the electron confinement around the Fermium nucleus, which results from the use of the Schrödinger equation, dramatically increases as |*Ę*_*n,1*_| increases. This strongly supports the substitution, in ab-initio software packages, of the Schrödinger’s radial wavefunctions for the ones which are solutions of Eq. (5), when simulations involving the inner electrons of heavy atoms should be conducted.

## Conclusions

In this work, first, it was obtained an exact analytical expression, which allows obtaining the quasi-relativistic energies of the bound states of the electron in Hydrogen-like atoms. The energies calculated in this way include the first-order perturbation relativistic correction to the kinetic energies calculated using the Schrödinger equation. Moreover, it was shown that Eq. (25) is the exact expression corresponding to the well-known approximate results given by Eq. (27). Second, it was discussed how to obtain the exact analytical wavefunctions of the quasi-relativistic wave equation used in this work (Eq. 5). The solutions were found following the same procedures, and with no more difficulty, than the ones present when solving the same problems using the Schrödinger equation. Nevertheless, the solutions found in this work are also valid when the particle is moving at energies as high as the energy associated to the particle’s mass. These quasi-relativistic atomic orbitals may be used for improving ab-initio software packages dedicated to numerical simulations in physical-chemistry and atomic and solid-state physics.
